# Health Care Costs, Utilization and Patterns of Care following Lyme Disease

**DOI:** 10.1371/journal.pone.0116767

**Published:** 2015-02-04

**Authors:** Emily R. Adrion, John Aucott, Klaus W. Lemke, Jonathan P. Weiner

**Affiliations:** 1 Department of Health Policy and Management, Johns Hopkins Bloomberg School of Public Health, Baltimore, Maryland, United States of America; 2 Johns Hopkins University School of Medicine, Division of Rheumatology, Johns Hopkins at Greenspring Station, 10755 Falls Road, Suite 200, Lutherville, Maryland, 21093, United States of America; 3 Department of Health Policy and Management, Johns Hopkins School of Public Health, Baltimore, Maryland, United States of America; 4 Department of Health Policy and Management, Johns Hopkins Bloomberg School of Public Health, Baltimore, Maryland, United States of America; University of North Dakota School of Medicine and Health Sciences, UNITED STATES

## Abstract

**Background:**

Lyme disease is the most frequently reported vector borne infection in the United States. The Centers for Disease Control have estimated that approximately 10% to 20% of individuals may experience Post-Treatment Lyme Disease Syndrome – a set of symptoms including fatigue, musculoskeletal pain, and neurocognitive complaints that persist after initial antibiotic treatment of Lyme disease. Little is known about the impact of Lyme disease or post-treatment Lyme disease symptoms (PTLDS) on health care costs and utilization in the United States.

**Objectives:**

1) to examine the impact of Lyme disease on health care costs and utilization, 2) to understand the relationship between Lyme disease and the probability of developing PTLDS, 3) to understand how PTLDS may impact health care costs and utilization.

**Methods:**

This study utilizes retrospective data on medical claims and member enrollment for persons aged 0-64 years who were enrolled in commercial health insurance plans in the United States between 2006-2010. 52,795 individuals treated for Lyme disease were compared to 263,975 matched controls with no evidence of Lyme disease exposure.

**Results:**

Lyme disease is associated with $2,968 higher total health care costs (95% CI: 2,807-3,128, p<.001) and 87% more outpatient visits (95% CI: 86%-89%, p<.001) over a 12-month period, and is associated with 4.77 times greater odds of having any PTLDS-related diagnosis, as compared to controls (95% CI: 4.67-4.87, p<.001). Among those with Lyme disease, having one or more PTLDS-related diagnosis is associated with $3,798 higher total health care costs (95% CI: 3,542-4,055, p<.001) and 66% more outpatient visits (95% CI: 64%-69%, p<.001) over a 12-month period, relative to those with no PTLDS-related diagnoses.

**Conclusions:**

Lyme disease is associated with increased costs above what would be expected for an easy to treat infection. The presence of PTLDS-related diagnoses after treatment is associated with significant health care costs and utilization.

## Introduction

Lyme disease is a growing health care problem in Northern Hemisphere countries worldwide, with cases in the United States increasing by approximately 200% in the last two decades. [1] [2] [3] Recent estimates indicate that the incidence of Lyme disease ranges from 240,000–440,000 new cases a year, making Lyme disease the seventh most common reportable infectious disease in the United States. [4] [5] The tick-borne bacteria associated with Lyme disease in North America, *Borrelia burgdorferi*, causes an early stage acute skin infection that often is associated with a skin lesion called erythema migrans. If left untreated, early Lyme disease may lead to neurologic and rheumatic manifestation weeks or months later. [6] Antibiotic treatment of Lyme disease is associated with more rapid resolution of early signs of infection and the prevention of the majority of the later, objective signs of disseminated infection.

Approximately 10–20% of patients treated for Lyme disease with a recommended 2–4 week course of antibiotics will have patient-reported symptoms that may last for weeks, months or years. [7] [8] Post-treatment Lyme disease symptoms have been described by numerous investigators and include fatigue, musculoskeletal pain, and neurocognitive complaints such as poor memory and concentration and extremity dysthesias. In some cases, symptoms may be severe, chronic and adversely affect health-related function. [8] [9] [10] [11] When post-treatment Lyme disease symptoms (PTLDS) persist for six months or longer and are associated with functional limitations in the patient, the illness has been termed "Post-treatment Lyme Disease Syndrome" by the Centers for Disease Control. [7] Because no sensitive biomarker for remotely treated Lyme disease exists, the true number of individuals at risk for the syndrome is unknown. [12] [13]

Although the frequency of Lyme disease has increased, the overall impact of Lyme disease and PTLDS in the United States has been difficult to ascertain using methods that rely on patient reports and reviews of medical records. [14] As a result, little is known about the impact of Lyme disease infection on health care utilization and costs. Although a few studies have examined the costs associated with Lyme disease, most have been small-scale studies, many of which have focused on Western European nations. [15] [16]

Moreover, of those studies that have looked at cost, the estimated impact of Lyme disease varies widely. Decision analysis models of Lyme disease treatment strategies have estimated the cost of acute uncomplicated Lyme disease to be less than $100 for the office visit, testing and antibiotic treatment. Late-stage Lyme disease with neurologic manifestation was estimated at $6,007. [17] [18] An early study from the Society of Actuaries that attempted to factor in PTLDS-related costs estimated the average cost of both uncomplicated and complicated cases to be approximately $10,000. [19] A Maryland study found that the annual direct medical cost of treating early- and late-stage Lyme disease decreased from means of $1,609 to $464 and $4,240 to $1,380, respectively, over the study period (1997–2000). [20] No attempt was made in this study to measure or account for PTLDS.

All of the above studies have been limited by a lack of access to large, nationally representative samples. The few studies that have used larger administrative databases have been limited in scope, focusing on cost of laboratory testing only. [21] [22] [23]

The association of PTLDS with Lyme disease and its significance remains controversial. Because of the limitations of the prior research in this area, the magnitude of health care utilization and costs associated with Lyme disease and PTLDS is currently unknown. The purpose of this study was threefold: first, to examine the impact of Lyme disease on 12-month health care costs and utilization, second, to understand the association between Lyme disease and risk of developing PTLDS, and third, to understand how PTLDS may impact 12-month health care costs and utilization.

## Methods

This study utilizes retrospective data on medical claims for persons enrolled in commercial health insurance plans. Individuals treated for Lyme disease were identified and compared to a matched sample of controls with no evidence of Lyme disease exposure.

### Data

**Fig 1:**
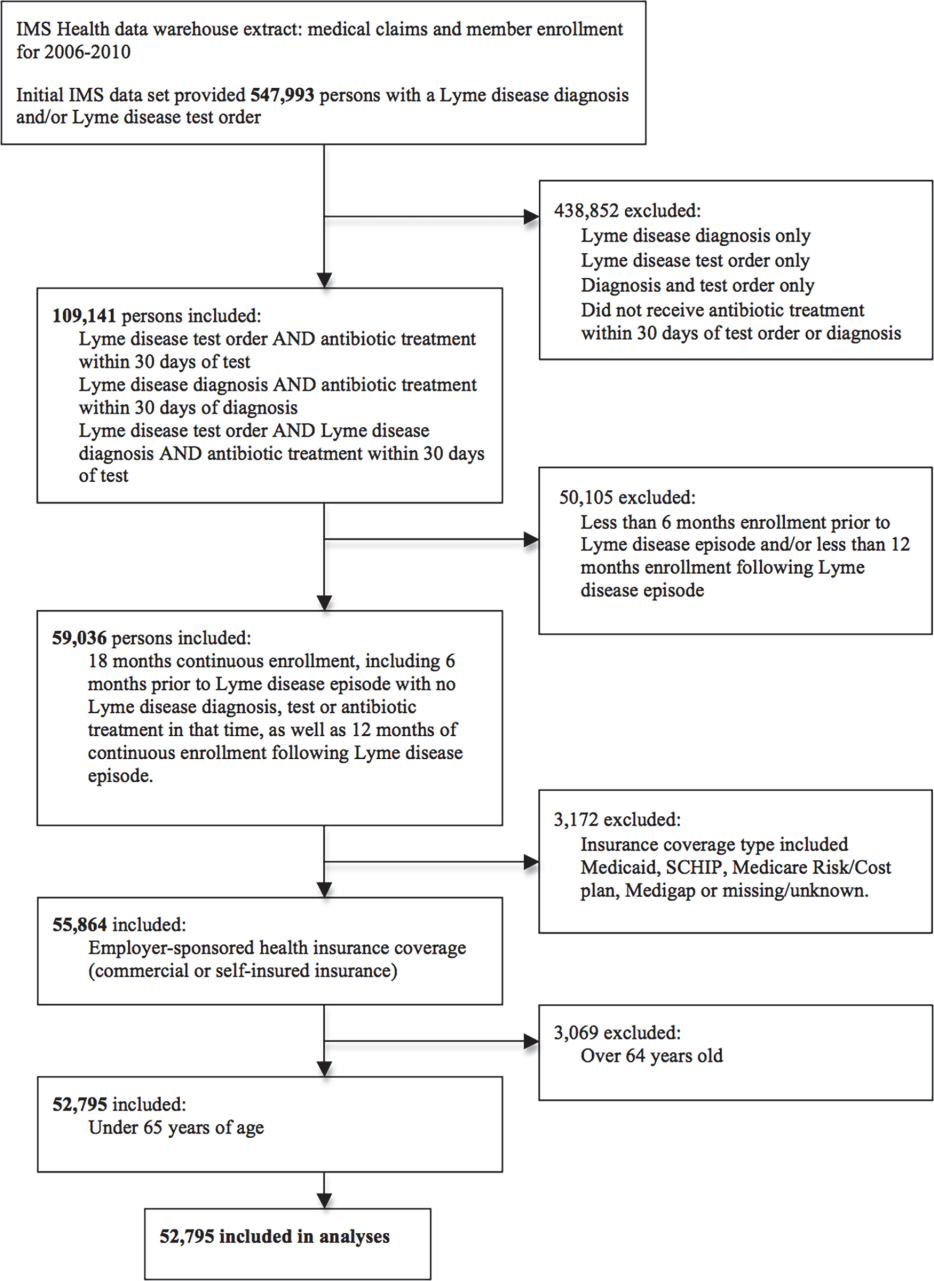
Lyme disease case selection flow diagram.

All data were drawn from the IMS Health LifeLink Health Plan Claims Database, which contains retrospective data on medical claims and member enrollment for approximately 47 million persons enrolled in a wide range of US commercial health insurance plans. The initial 547,993 potential Lyme disease cases were selected based on presence of a Lyme disease diagnosis and/or test order between 2006–2010 [Fig. 1]. To increase the specificity of our identification of Lyme disease cases, we narrowed the case identification to include only those individuals with antibiotic treatment within 30 days of either a Lyme disease test order (CPT code 86618) and/or a Lyme disease diagnosis (ICD code 088.81). Of the original 547,993 persons with a Lyme disease test order and/or diagnosis, 109,141 (19.9%) received antibiotics appropriate for the treatment of Lyme disease within 30 days of that test order/diagnosis.

We further narrowed our sample to include only those individuals with 18 months of continuous enrollment in a health plan, including 6 months of enrollment prior to their Lyme disease episode as a “clean period” in which they were neither tested for, nor diagnosed with, Lyme disease. We excluded a relatively small number for whom insurance coverage was from any source other than a commercial or self-insured employer-sponsored health insurance plan, and individuals over 64 years of age. The final number of Lyme disease cases that met the above criteria totaled 52,795.

The control group was also drawn from the IMS database and included only those individuals with no Lyme disease test orders or diagnoses, at least 18 months of continuous enrollment, and at least one paid outpatient claim. The control group was selected by performing a 5-to-1 matching of controls to cases using stratified random sampling without replacement, matching on age, sex, enrollment year, region, and payer type (commercial or self-insured). The final number of controls included in the analysis totaled 263,975. 12-month health care costs and utilization for the control group are measured from January 1 of the matched year.

This study was ruled to be not human subjects research (NHSR) and thus IRB exempt by the Johns Hopkins School of Medicine Internal Review Board on 10/19/2011. The data utilized constitute fully de-identified data; the research file has an encrypted identifier and does not include patient names, addresses, or zip codes.

### Outcome measures

We analyzed 11 measures of health care costs: total costs, total inpatient, total pharmacy, total outpatient, outpatient anesthesiology, outpatient evaluation and management, outpatient medicine, outpatient pathology laboratory, outpatient radiology, outpatient surgery, and all other outpatient costs. Current Procedure Terminology (CPT) codes were used to group outpatient costs by type of outpatient service. All cost variables are measured as the “allowed” amount as defined by the insurer and represent the cost summed over each member’s 12-month in-scope period.

There are two measures of utilization, chosen because we hypothesized these categories would be most likely to be affected by a Lyme disease diagnosis: outpatient management and evaluation visits, and emergency department visits. Each utilization measure equals the total number of visits in that category in a 12-month period. The Johns Hopkins Adjusted Clinical Groups (ACG) System was used to measure morbidity burden and utilization. [24]

Based on the Expanded Diagnosis Clusters (EDCs) of the ACG case-mix system, groupings of diagnosis codes associated with PTLDS were identified by a panel of clinicians familiar with Lyme disease. The 5 relevant EDCs identified were examined and any International Classifications of Diseases (ICD) codes within each EDC that were not associated with PTLDS were excluded. The 5 EDC categories containing only those PTLDS-related ICD codes used in the analysis include: debility and undue fatigue, musculoskeletal signs and symptoms, peripheral neuropathy/neuritis, arthropathy, and non-specific signs and symptoms. The analyses presented here utilize these predominantly symptom-based diagnostic codes, as objective biomarkers for the diagnosis of post-treatment Lyme disease syndrome are not available. [12] Each EDC category was converted into a binary variable indicating whether that individual had any claims in that category during the study period.

### Control variables

The adjusted analyses presented here control for age, sex, region and enrollment year. In addition, the analyses control for a number of conditions associated with high costs and utilization. A clinical team identified 44 high cost conditions not related to Lyme disease by reviewing a complete list of EDCs created by the Johns Hopkins ACG system. A list of the 44 high cost conditions identified is available from the authors upon request.

### Cost and utilization analyses

Unadjusted means for each category of cost and utilization were determined for both cases and controls and Welch’s t-tests were used to assess differences in means between the groups. Generalized linear models (GLM) were used to examine the relationship between Lyme disease status and cost. GLM models can account for non-normality in data distribution while still allowing for inferences about mean costs. The identity link and Gaussian distribution were utilized for all generalized linear model analyses.

Negative binomial regression analysis, a technique commonly used with count data that is over-dispersed, was used to examine the adjusted impact of Lyme disease status on health care utilization. Huber/White sandwich estimators were used to determine the standard errors and p-values.

### Health outcomes analyses

Unadjusted frequencies for each PTLDS-related EDC category were calculated for both groups and chi-square tests were conducted to assess whether there were statistically significant differences. Multivariable logistic regression analysis was used to examine the impact of Lyme disease on the odds of PTLDS-related diagnoses.

### PTLDS cost and utilization analyses

The above analyses were repeated to compare cost and utilization outcomes in the Lyme disease group for those with 1 or more PTLDS-related claims relative to those with no PTLDS-related claims. All analyses presented here were completed using STATA v. 10.

## Results

### Demographics of the samples

The distributions of age, sex and region of the Lyme disease cases in our sample are consistent with what one would expect given national statistics on Lyme disease [Table 1]. Both cases and controls are approximately 51% female. Nearly half of both groups are aged 45–64 years, with 31% between ages 21–44 and the remaining 19% under 20 years of age. The vast majority of those in our study—approximately 80%—reside in highly endemic geographic regions, including the Northeast, Mid-Atlantic and Great Lakes regions [Table 1, S1 Table]. The smaller number of cases in other regions may be due to travel exposure or to the possibility of low rates of transmission from ixodes scapularis vectors in southeastern states. This hypothesis is strengthened by the fact that the majority of our “non-endemic” cases came from the southeast and Appalachian regions and not from the central, plains or desert regions. The seasonal diagnosis of Lyme disease among our sample peaked in June and July, with a nadir in January and February [S1 Fig.], consistent with national statistics for Lyme disease.

**Table 1:** Characteristics of study sample.

	**Lyme disease sample** * ^,^ ^†^	Matched controls^‡^
	No. (%)	No. (%)
**Total**	52,795 (100)	263,975 (100)
		
**Sex**		
Female	27,138 (51.4)	135,690 (51.4)
Male	25,657 (48.6)	128,285 (48.6)
		
**Age**		
0–4 years	1,176 (2.2)	5,880 (2.2)
5–9 years	2,722 (5.2)	13,610 (5.2)
10–20 years	6,713 (12.7)	33,565 (12.7)
21–44 years	16,182 (30.7)	80,910 (30.7)
45–64 years	26,002 (49.3)	130,010 (49.3)
		
**Region**		
Northeast	19,921 (37.7)	99,605 (37.7)
Mid-Atlantic	12,553 (23.8)	62,765 (23.8)
Great Lakes	9,587 (18.2)	47,935 (18.2)
Appalachian	4,145 (7.9)	20,735 (7.9)
Southeast	3,927 (7.4)	19,635 (7.4)
Central	1,534 (2.9)	7,670 (2.9)
Pacific	561 (1.1)	2,805 (1.1)
Plains & Mountains	321 (0.6)	1,605 (0.6)
Desert	246 (0.5)	1,230 (0.5)
		

^*^ Lyme disease sample includes only those persons with a test order and antibiotic treatment within 30 days of the test order, a diagnosis and antibiotic treatment within 30 days of the diagnosis, or a diagnosis, test order and antibiotic treatment within 30 days. The Lyme disease sample includes only those with 18 consecutive months of enrollment, including a 6-month “clean period” of enrollment prior to Lyme disease episode in which they were neither diagnosed with nor tested for Lyme disease.

^†^ Lyme disease and control samples are restricted to persons under 65 years of age, with commercial health insurance plans.

^**‡**^ Controls were matched to Lyme disease cases on age, sex, region, payer and enrollment year. Control group includes only those with 12 consecutive months of enrollment in a commercial health insurance plan. Control group was restricted to persons with outpatient costs greater than $0.

### Cost and utilization analyses

Columns I and II of Table 2 show that, in most categories, unadjusted mean 12-month costs are up to two times higher for the Lyme disease cases as compared to the controls, and the unadjusted mean number of visits over a 12-month period were higher for the cases as compared to controls for both measures of utilization. Adjusted analyses (Column IV) show that Lyme disease is associated with $2,968 higher total health care costs (95% CI: 2,807–3,128, p<.001), $464 higher outpatient evaluation and management costs (95% CI: 456–472, p<.001) and $612 higher pharmacy costs (95% CI: 580–645, p<.001) over a 12-month period, as compared to the control group. Lyme disease is associated with 87% higher evaluation and management visits (95% CI: 86%-89%, p<.001) and 71% higher emergency department visits (95% CI: 68%-76%, p<.001) over a 12-month period, relative to the control group.

**Table 2:** 12-month health care costs and utilization, Lyme disease and control groups.

		(I) **Unadjusted mean during 12-month study period, Lyme disease group** * ^,^ ^†^	(II) **Unadjusted mean during 12-month study period, control group** ^†^ ^,^ ^**‡**^	(III) **Welch’s t-test** (of difference in means)	(IV) **Adjusted impact of Lyme disease diagnosis on 12-month health care costs and utilization, (Robust SE)**
					**Adjusted impact of Lyme disease diagnosis on 12-month health care costs (Robust SE)** ^||^
Total cost		$ 8,205	$ 4,421	p<.001	$ 2,968 (81.9)***
Inpatient cost		$ 1,710	$ 1,038	p<.001	$ 230 (55.9)***
Pharmacy cost		$ 1,525	$ 826	p<.001	$ 612 (16.6)***
Outpatient cost		$ 4,969	$ 2,557	p<.001	$ 2,125 (47.4)***
Cost by outpatient category:	Evaluation and Management	$ 904	$ 412	p<.001	$ 464 (4.1)***
	**Medicine** ^§^	$ 622	$ 326	p<.001	$ 275 (7.4)***
	Pathology Laboratory	$ 487	$ 140	p<.001	$ 332 (4.4)***
	Radiology	$ 575	$ 244	p<.001	$ 294 (9.0)***
	Anesthesiology	$ 100	$ 58	p<.001	$ 38 (1.6)***
	Surgery	$ 605	$ 328	p<.001	$ 255 (8.4)***
	Other (none of the above)	$ 1,677	$ 1,048	p<.001	$ 467 (36.2)***
					
					**Adjusted impact of Lyme disease diagnosis on 12-month health care utilization, (Robust SE)** ^¶^
Outpatient management and evaluation visits		6.97	3.65	p<.001	1.87 (0.008)***
Emergency visits		0.35	0.20	p<.001	1.71 (0.020)***
					

*** statistically significant at p<.001 level,

^*^ Lyme disease sample includes only those persons with a test order and antibiotic treatment within 30 days of the test order, a diagnosis and antibiotic treatment within 30 days of the diagnosis, or a diagnosis, test order and antibiotic treatment within 30 days. The Lyme disease sample includes only those with 18 consecutive months of enrollment, including a 6-month “clean period” of enrollment prior to Lyme disease episode in which they were neither diagnosed with nor tested for Lyme disease.

^†^ Lyme disease and control samples are restricted to persons under 65 years of age, in commercial health insurance plans.

^**‡**^ Controls were matched to Lyme disease cases on age, sex, region, payer and enrollment year. Control group includes only those with 18 consecutive months of enrollment in a commercial health insurance plan. Control group was restricted to persons with outpatient costs greater than $0.

^§^ Medicine includes acupuncture, home health, home infusion and other special services, procedures and reports.

^||^ Adjusted impact calculations based on GLM regression analysis using the Huber/White sandwich estimator of variance and adjusting for year, region, age, and sex, and controlling for 44 high-cost conditions

^¶^ Adjusted ratio calculations for outpatient and emergency visits are based on negative binomial regression analysis using the Huber/White sandwich estimator of variance and adjusting for year, region, age, and sex, and controlling for 44 high-cost conditions.

The adjusted analyses presented here control for a number of factors, including a variety of medical conditions that can result in high costs and greater utilization of health care. The differences between the findings of the unadjusted and adjusted analyses are likely due, in part, to differing rates of these conditions between the controls and cases.

### Post-treatment Lyme disease symptom analyses

Table 3 shows that the unadjusted frequencies of various categories of post-treatment Lyme disease symptom (PTLDS)-related diagnoses are higher and statistically different across all categories for the Lyme disease group relative to the control group.

**Table 3:** Post-treatment Lyme disease symptom-related diagnoses, Lyme disease and control groups.

	(I) **Frequency of PTLDS-related diagnoses, Lyme disease group** ^*^ ^,^ ^**‡**^	(II) **Frequency of PTLDS-related diagnoses, control group** ^†^ ^,**‡**^	(III) **χ** ^**2**^ **test** (of difference in frequencies)	(IV) **Adjusted odds of PTLDS-related diagnoses for Lyme disease group versus control group, (Robust SE)** ^§^
Any PTLDS-related diagnosis	63.1%	27.6%	p<.001	4.77 (0.05)***
Any PTLDS-related diagnosis, females	68.3%	30.1%	p<.001	5.38 (0.08)***
Any PTLDS-related diagnosis, males	57.5%	24.9%	p<.001	4.24 (0.06)***
				
Debility and undue fatigue	32.7%	8.4%	p<.001	5.47 (0.07)***
Non-specific signs and symptoms: memory loss, acute/chronic pain	3.8%	1.2%	p<.001	3.10 (0.09)***
Musculoskeletal signs and symptoms	45.2%	18.9%	p<.001	3.62 (0.04)***
Arthropathy	7.7%	1.8%	p<.001	4.51 (0.10)***
Peripheral neuropathy, neuritis	11.4%	4.6%	p<.001	2.65 (0.05)***
				

*** statistically significant at p<.001 level

^*^ Lyme disease sample includes only those persons with a test order and antibiotic treatment within 30 days of the test order, a diagnosis and antibiotic treatment within 30 days of the diagnosis, or a diagnosis, test order and antibiotic treatment within 30 days. The Lyme disease sample includes only those with 18 consecutive months of enrollment, including a 6-month “clean period” of enrollment prior to Lyme disease episode in which they were neither diagnosed with nor tested for Lyme disease.

^†^ Controls were matched to Lyme disease cases on age, sex, region, payer and enrollment year. Control group includes only those with 18 consecutive months of enrollment in a commercial health insurance plan. Control group was restricted to persons with outpatient costs greater than $0.

^**‡**^ Lyme disease and control samples are restricted to persons under 65 years of age, with commercial health insurance plans.

^§^ Odds are calculated using logistic regression analysis and are adjusted for year, region, age, and sex, and controls for 44 high-cost conditions.

Adjusted analyses show that Lyme disease is associated with 4.77 times greater odds of having one or more PTLDS-related diagnosis (95% CI: 4.67–4.87, p<.001). Persons in the Lyme disease group had 5.47 times greater odds of experiencing debility and undue fatigue (95% CI: 5.35–5.60, p<.001), 2.6 times greater odds of experiencing peripheral neuropathy (95% CI: 2.57–2.74, p<.001) and 4.5 times greater odds of experiencing arthropathy (95% CI: 4.32–4.70, p<.001).

Table 4 presents our analyses of the impact within the Lyme disease group of PTLDS-related conditions on 12-month costs and utilization. Unadjusted mean costs in all categories were twice as high, and the mean number of evaluation and management visits was higher for those with one or more PTLDS-related diagnosis, as compared to those with no PTLDS-related diagnoses (columns I and II). Adjusted analyses show that, among the Lyme disease group, having one or more PTLDS-related diagnosis was associated with $3,798 higher total costs (95% CI: 3,542–4,055, p<.001), 66% more outpatient evaluation and management visits (95% CI: 64%-69%, p<.001), and 89% more emergency department visits (95% CI: 81%-97%, p<.001) over a 12-month period, relative to those with no PTLDS-related diagnoses (column IV).

**Table 4:** Adjusted 12-month health care costs and utilization for patients in the Lyme disease sample with one or more post-treatment Lyme disease symptom (PTLDS)-related diagnosis, as compared to patients in Lyme disease sample with no post-treatment Lyme disease symptom-related diagnoses.

	(I) **Mean, Lyme disease group with no PTLDS-related diagnoses**	(II) **Mean, Lyme disease group with 1 or more PTLDS-related diagnosis**	(III) **Welch’s t-test** (of difference in means)	(IV) **Adjusted impact on costs and utilization of having one or more PTLDS-related diagnosis, as compared to no PTLDS-related diagnoses, among those in the Lyme disease group**
				**Adjusted impact of one or more PTLDS-related diagnosis on 12-month health care costs, relative to no PTLDS-related diagnoses, (Robust SE)** ^*^
Total cost	$ 4,418	$ 10,423	p<.001	$ 3,798 (131)***
Outpatient cost	$ 2,570	$ 6,375	p<.001	$ 2,786 (74)***
Inpatient cost	$ 905	$ 2,181	p<.001	$ 443 (88)***
Pharmacy cost	$ 942	$ 1,867	p<.001	$ 569 (30)***
				
				**Adjusted ratio of visits for those with 1 or more PTLDS-related diagnosis relative to those with no PTLDS-related diagnoses, (Robust SE)** ^†^
Outpatient management and evaluation visits	4.55	8.39	p<.001	1.66 (0.01)***
Emergency visits	0.22	0.43	p<.001	1.89 (0.02)***
				

Note: Above table includes Lyme disease sample only. Lyme disease sample includes only those persons with a test order and antibiotic treatment within 30 days of the test order, a diagnosis and antibiotic treatment within 30 days of the diagnosis, or a diagnosis, test order and antibiotic treatment within 30 days. The Lyme disease sample includes only those with 18 consecutive months of enrollment, including a 6-month “clean period” of enrollment prior to Lyme disease episode in which they were neither diagnosed with nor tested for Lyme disease. Lyme disease sample restricted to persons under 65 years of age, with commercial health insurance plans

^*^ Adjusted impact calculations based on GLM regression analysis using the Huber/White sandwich estimator of variance and adjusting for year, region, age, and sex, and controlling for high-cost non-Lyme disease-related conditions.

^†^ Adjusted ratio calculations for outpatient and emergency visits are based on negative binomial regression analysis using the Huber/White sandwich estimator of variance and adjusting for year, region, age, and sex, and controlling for high-cost non-Lyme disease-related conditions.

As with all analyses, in order for the above results to hold we assume minimal unmeasured confounding.

## Discussion

Treatment of Lyme disease is known to be effective in preventing further complications such as meningitis and joint arthritis. However, the impact on health care utilization of symptoms that may persist after antibiotic treatment is unknown. [12]. The significance of persistent symptoms after completion of standard antibiotic therapy of Lyme disease is a controversial topic. At the core of the controversy is how common, severe and prolonged are post-treatment Lyme symptoms and how individuals should be diagnosed and treated when PTLDS is suspected. Assumptions used for cost effectiveness analysis have assumed a single visit would be necessary for early cutaneous disease,[18] however our data show that Lyme disease was associated with 87% more evaluation and management visits and 71% more emergency department visits as compared to controls. This increased utilization seems at odds with the community standard of care and the Infectious Disease Society Guidelines for the treatment of Lyme disease, which do not call for follow up visits to document response to treatment in early Lyme disease. [12]

Mean total costs for individuals with Lyme disease were almost twice those of controls, and adjusted total costs were $2,968 greater. These figures are significantly higher than the $100 predicted costs of treating uncomplicated Lyme disease used in previous theoretical models and higher than the $464-$1,609 mean costs of treating early stage Lyme disease reported by Zhang et al. [20] This may reflect the importance of accounting for utilization and costs associated with PTLDS. Moreover, the especially large difference in laboratory costs between the groups suggests that physicians may be attempting to understand persistent symptoms experienced by individuals with PTLDS through further diagnostic testing.

Over 63% of the Lyme disease cases had at least one diagnosis associated with PTLDS, which is 36 percentage points higher a rate than the prevalence of the same symptoms in our control population [Table 3]. Our estimates are higher than the CDC’s estimated rate of Post-Treatment Lyme Disease Syndrome of 10–20% in part because we examined the proportion of people with *any* PTLDS-related diagnosis, without requiring a demonstrated functional decline, which was not captured in this study. The difference may also reflect a greater complexity of our community-based sample and increased risk factors such as delayed diagnosis in our population compared to the more uniform populations of ideally diagnosed and treated patients studied in prospective cohorts in the literature.

Fatigue is one of the central features of PTLDS. In our study, persons with Lyme disease were 5.5 times more likely to have a diagnosis of debility and undue fatigue, which suggests that fatigue after treatment of Lyme disease is more frequent than in the general population. Our data are limited by the inability to measure the severity of fatigue, however other studies have shown that fatigue can be severe and impact the health-related quality of life of patients. [25]

Arthropathy and peripheral neuropathy also stood out, with cases having 4.5 and 2.6 greater odds of these diagnoses, respectively, as compared to controls. The relative infrequency of these diagnoses in the general population supports them as more specific markers of PTLDS. The diagnostic codes used for arthropathy and neuropathy cannot easily distinguish diagnoses based on symptoms versus clinical diagnoses confirmed by objective signs or laboratory testing. However, the increased frequency of these diagnostic codes among cases suggests that musculoskeletal and neurologic conditions are more common than among our controls.

Sensitivity analyses were performed, looking at diagnoses that would not be expected to be related to Lyme disease. Diagnoses such as diabetes were found to be more prevalent in the control population than in the Lyme disease group [S2 Table].

The analyses presented here also indicate that PTLDS-related diagnoses are associated with notably higher utilization and costs among those with Lyme disease. Having one or more PTLDS-related diagnosis was associated with 66% more outpatient evaluation and management visits and adjusted 12-month total costs that were $3,798 higher as compared to those with Lyme disease with no PTLDS-related diagnoses.

### Limitations

While an attempt was made to use a conservative case definition, it was not possible to examine medical records to confirm each case of Lyme disease. However, the specificity of our Lyme disease case definition was increased by including only the 20% of individuals who had antibiotic therapy prescribed following a diagnostic test order. Our percentage of “antibiotic confirmed” tests is similar to a recent report that 10–19% of Lyme disease tests performed in commercial labs are positive. [4] In addition, the geographic and seasonal characteristics of our cohort conform closely to the known epidemiology of Lyme disease. If the cohort were contaminated with patients being treated for illnesses incorrectly attributed to Lyme disease such as fibromyalgia, chronic fatigue syndrome or patients with medically unexplained symptoms, we would expect to see the markedly higher female to male ratio characteristic of those diagnoses, which was not observed.

Costs for the Lyme disease group might be expected to be higher, particularly if expensive long term intravenous antibiotics were used, a practice that has been reported in the treatment of the more heterogeneous and complex group of patients with long term chronic symptoms where Lyme disease may not be the sole cause of illness. [26] However, we did not find increased costs associated with IV antibiotics in our study population [S3 and S4 Tables].

Our study sample included only persons under 65 years of age who were continuously enrolled in employer-sponsored health insurance plans for 18 months, thus, our sample may not be representative of all Lyme disease cases in the United States. In addition, our analyses only looked at costs over a 12-month period, so we were unable to capture any longer-term costs associated with Lyme disease or PTLDS, nor were our analyses able to account for any indirect costs or costs associated with lost productivity due to Lyme disease, which may be significant. [27]

A key concern of ours was the degree to which PTLDS-related diagnoses may have been present prior to infection with Lyme disease. Among the Lyme disease group, 21,753 persons had no PTLDS-related diagnoses, 14,470 persons had one or more PTLDS-related diagnosis post-infection only, 9,308 persons had PTLDS-related diagnoses both pre- and post-infection and 7,264 had PTLDS-related diagnoses pre-infection only. Ultimately, we decided that a comparison between existing diagnoses for the Lyme disease group and existing diagnoses in the control group was a better “apples-to-apples” comparison. However, sensitivity analyses were performed comparing new (post-infection only) PTLDS-related diagnoses among the Lyme disease group to existing diagnoses among the control group [S5 and S6 Tables]. These analyses showed that, with the exception of musculoskeletal signs and symptoms, the Lyme disease group had less dramatic but still greater adjusted odds of all PTLDS-related diagnoses as compared to the control group.

### Conclusions

Lyme disease and the ongoing symptoms that may occur after initial antibiotic treatment represent a significant source of health care utilization and costs. These increased costs may have a considerable impact on overall health care spending in the United States. Extrapolating from the data, if we assume that, per the estimates released by Hinckley et al., there are approximately 240,000–440,000 cases of Lyme disease annually [4], and using our estimate of $2,968 greater annual health care costs for those diagnosed with Lyme disease, the total direct medical costs attributable to Lyme disease and PTLDS could be somewhere between $712 million - $1.3 billion each year. As the number of cases increases, the future economic impact of Lyme disease would be expected to increase.

The public health policy implications of this study are significant, particularly with the high number of cases in endemic regions and the potential for continued spread into new regions of the United States. Increased awareness of the potential complications associated with Lyme disease is crucial in the primary care and consultative settings to avoid misdiagnosis and unnecessary diagnostic testing. Public health and medical guidelines need to move beyond the debate over whether “chronic Lyme disease” exists, as, regardless of one’s perspective, these patients do demonstrate increased health care costs and utilization. Effective, cost-effective, and compassionate management of this group of patients is essential to decrease costs and improve outcomes even in the absence of a definitive understanding of the pathophysiology of this post-treatment illness.

## Supporting Information

S1 Fig Distribution of Lyme disease sample by month of diagnosis (PDF)

S1 Table Regions and States. (PDF)

S2 Table Adjusted odds of non-PTLDS-related diagnoses for Lyme disease group versus control group. (PDF)

S3 Table Type of antibiotic prescribed, Lyme disease sample. (PDF)

S4 Table Home infusion analyses. (PDF)

S5 Table Post-treatment Lyme disease-related condition presence by time of diagnosis (pre-Lyme disease and/or post-Lyme disease period), Lyme disease group. (PDF)

S6 Table Post-treatment Lyme disease symptom-related diagnoses among Lyme disease and control groups, using diagnoses recorded post-Lyme disease episode only for Lyme disease group. (PDF)
