# Supplementary material for: Health Care Costs, Utilization and Patterns of Care following Lyme Disease
Source: PLoS One. 2015 Feb 4;10(2):e0116767. doi: 10.1371/journal.pone.0116767 (PMC4317177; doi:10.1371/journal.pone.0116767)
Supplement: S1 Fig — (PDF) [file pone.0116767.s001.pdf]

Figure S1. Distribution of Lyme disease sample by month of diagnosis*

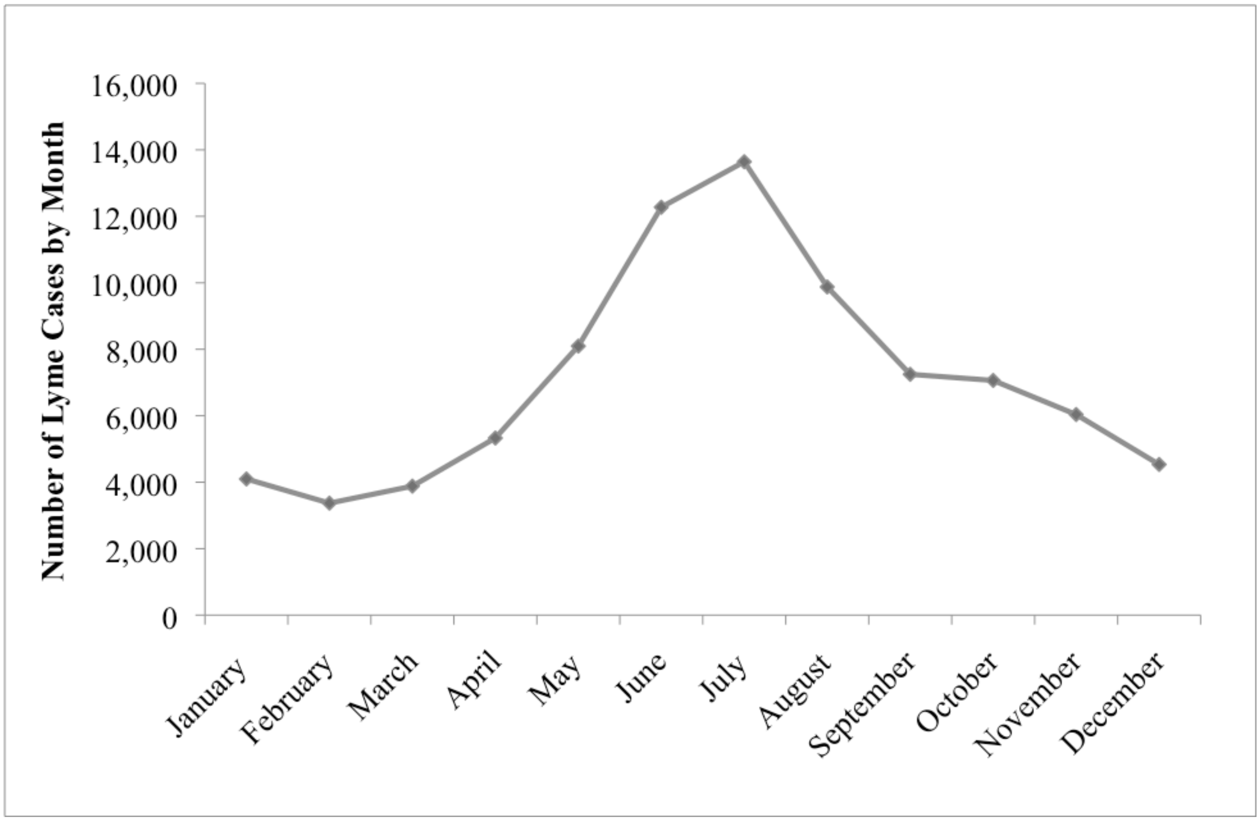

* "Month of diagnosis" defined as the month in which an individual's claims indicated they received a Lyme disease test order and antibiotic treatment, a Lyme disease diagnosis and antibiotic treatment, or a Lyme disease diagnosis, test order and antibiotic treatment.
