# Supplementary material for: Health Care Costs, Utilization and Patterns of Care following Lyme Disease
Source: PLoS One. 2015 Feb 4;10(2):e0116767. doi: 10.1371/journal.pone.0116767 (PMC4317177; doi:10.1371/journal.pone.0116767)
Supplement: S1 Table — (PDF) [file pone.0116767.s002.pdf]

Table S1. Regions and States

Northeast	Mid-Atlantic	Great Lakes	Pacific	Southeast
Maine	Pennsylvania	Minnesota	Washington	N. Carolina
Vermont	New Jersey	Michigan	Oregon	S. Carolina
New Hampshire	Delaware	Wisconsin	California	Georgia
Connecticut	Maryland	Illinois	Alaska	Florida
Massachusetts	Virginia	Iowa	Hawaii	Alabama
Rhode Island	D.C.	Indiana		
New York				
**Appalachian**	**Central**	**Plains/Mt.**	**Desert**	
Ohio	Missouri	N. Dakota	Arizona	
W. Virginia	Arkansas	S. Dakota	New Mexico	
Tennessee	Texas	Wyoming	Utah	
Kentucky	Oklahoma	Nebraska	Colorado	
Mississippi	Louisiana	Kansas	Nevada	
		Idaho		
		Montana		
