# Supplementary material for: Health Care Costs, Utilization and Patterns of Care following Lyme Disease
Source: PLoS One. 2015 Feb 4;10(2):e0116767. doi: 10.1371/journal.pone.0116767 (PMC4317177; doi:10.1371/journal.pone.0116767)
Supplement: S2 Table — (PDF) [file pone.0116767.s003.pdf]

Table S2. Adjusted odds of non-PTLDS-related diagnoses for Lyme disease group versus control group

Sensitivity analyses looking at diagnoses that would not be expected to be related to Lyme disease.

	Adjusted odds of assorted diagnoses for Lyme disease group versus control group*	p-value
**Cough**	1.61	p<.001
**Allergic rhinitis**	1.5	p<.001
**Sinusitis**	1.85	p<.001
**Type I diabetes w/o complications**	0.82	p=.015
**Type II diabetes w/o complications**	0.77	p<.001
**Asthma, w/o status asthmaticus**	1.4	p<.001
**Congestive heart failure**	0.86	p=.052
**Malignant neoplasms, lung**	0.85	p=.303

* Odds ratios were calculated using logistic regression analysis and are adjusted for year, region, age, and sex, and controls for 44 high-cost conditions.
Lyme disease sample includes only those persons with a test order and antibiotic treatment within 30 days of the test order, a diagnosis and antibiotic treatment within 30 days of the diagnosis, or a diagnosis, test order and antibiotic treatment within 30 days. The Lyme disease sample includes only those with 18 consecutive months of enrollment, including a 6-month “clean period” of enrollment prior to Lyme disease episode in which they were neither diagnosed with nor tested for Lyme disease.
Controls were matched to Lyme disease cases on age, sex, region, payer and enrollment year. Control group includes only those with 18 consecutive months of enrollment in a commercial health insurance plan. Control group was restricted to persons with outpatient costs greater than $0.
Lyme disease and control samples are restricted to persons under 65 years of age, with commercial health insurance plans.
