# Supplementary material for: Health Care Costs, Utilization and Patterns of Care following Lyme Disease
Source: PLoS One. 2015 Feb 4;10(2):e0116767. doi: 10.1371/journal.pone.0116767 (PMC4317177; doi:10.1371/journal.pone.0116767)
Supplement: S3 Table — (PDF) [file pone.0116767.s004.pdf]

Table S3. Type of antibiotic prescribed, Lyme disease sample*

Antibiotic	No. (%)†
Missing	62 (0.12)
Amoxicillin	10,115 (19.16)
Amoxicillin-clavulanate	4,533 (8.59)
Cefotaxime	67 (0.13)
Ceftriaxone	2,658 (5.03)
Cefuroxime	2,241 (4.24)
Doxycycline	31,705 (60.05)
Erythromycin	316 (0.6)
Penicillin	771 (1.46)
Tetracycline	327 (0.62)

* Lyme disease sample includes only those persons with a test order and antibiotic treatment within 30 days of the test order, a diagnosis and antibiotic treatment within 30 days of the diagnosis, or a diagnosis, test order and antibiotic treatment within 30 days. The Lyme disease sample includes only those with 18 consecutive months of enrollment, including a 6-month "clean period" of enrollment prior to Lyme disease episode in which they were neither diagnosed with nor tested for Lyme disease.

† Out of total 52,795 cases included in Lyme disease sample.
