# Supplementary material for: Health Care Costs, Utilization and Patterns of Care following Lyme Disease
Source: PLoS One. 2015 Feb 4;10(2):e0116767. doi: 10.1371/journal.pone.0116767 (PMC4317177; doi:10.1371/journal.pone.0116767)
Supplement: S4 Table — (PDF) [file pone.0116767.s005.pdf]

Table S4. Home infusion analyses

Costs associated with antibiotic treatment do not make up a substantial percentage of the overall costs in the patients with Lyme disease. As seen in Appendix Table 3, a small percentage, 5%, of our population received intravenous ceftriaxone as their initial therapy, presumably for neurologic or cardiac involvement with Lyme disease. In the supplemental analysis presented below, we found that the cost of intravenous therapy averages out to be a small cost in the study population, though the cost of intravenous therapy may represent a substantial cost in a given individual.

	**Lyme disease group***	**Control group†**
**Unadjusted mean 12-month home infusion spending (Range)**	$11.83 ($0 - $28,416)	$0.66 ($0 - $5,568)
**No. of persons with any home infusion spending (%)**	761 (1.4)	254 (0.1)
**Unadjusted mean 12-month home infusion spending among those with any home infusion spending (Range)**	$820 ($65 - $28,416)	$690 ($70 - $5,568)
**Adjusted impact of Lyme disease on 12-month home infusion costs‡**	$9.72***	

*** = p<.001

* Lyme disease sample includes only those persons with a test order and antibiotic treatment within 30 days of the test order, a diagnosis and antibiotic treatment within 30 days of the diagnosis, or a diagnosis, test order and antibiotic treatment within 30 days. The Lyme disease sample includes only those with 18 consecutive months of enrollment, including a 6-month "clean period" of enrollment prior to Lyme disease episode in which they were neither diagnosed with nor tested for Lyme disease.

† Controls were matched to Lyme disease cases on age, sex, region, payer and enrollment year. Control group includes only those with 18 consecutive months of enrollment in a commercial health insurance plan. Control group was restricted to persons with outpatient costs greater than $0.

‡ Calculation based on GLM regression analysis and adjusting for year, region, age, and sex, and controlling for 44 high-cost non-Lyme disease-related conditions.
