# Supplementary material for: Health Care Costs, Utilization and Patterns of Care following Lyme Disease
Source: PLoS One. 2015 Feb 4;10(2):e0116767. doi: 10.1371/journal.pone.0116767 (PMC4317177; doi:10.1371/journal.pone.0116767)
Supplement: S5 Table — (PDF) [file pone.0116767.s006.pdf]

Table S5. Post-treatment Lyme disease-related condition presence by time of diagnosis (pre-Lyme disease and/or post-Lyme disease period), Lyme disease group*

	Person Counts by Condition Presence in Pre and/or Post Lyme Period			
**Condition/EDC Category**†	**Count of selected ICD Codes**	**Persons with Condition Pre Lyme Only**	**Persons with Condition Pre and Post Lyme**	**Persons with Condition Post Lyme Only**
**Arthropathy**	82	1,087	273	2,336
**Debility and undue fatigue**	3	3,918	1,192	6,612
**Musculoskeletal signs and symptoms**	41	5,198	1,826	8,753
**Nonspecific signs and symptoms**	8	588	127	1,300
**Peripheral neuropathy, neuritis**	72	1,685	669	3,301

* Lyme disease sample includes only those persons with a test order and antibiotic treatment within 30 days of the test order, a diagnosis and antibiotic treatment within 30 days of the diagnosis, or a diagnosis, test order and antibiotic treatment within 30 days. The Lyme disease sample includes only those with 18 consecutive months of enrollment, including a 6-month "clean period" of enrollment prior to Lyme disease episode in which they were neither diagnosed with nor tested for Lyme disease.

† A person may have claims in multiple EDC categories.
