# Supplementary material for: Health Care Costs, Utilization and Patterns of Care following Lyme Disease
Source: PLoS One. 2015 Feb 4;10(2):e0116767. doi: 10.1371/journal.pone.0116767 (PMC4317177; doi:10.1371/journal.pone.0116767)
Supplement: S6 Table — (PDF) [file pone.0116767.s007.pdf]

Table S6. Post-treatment Lyme disease symptom-related diagnoses among Lyme disease and control groups, using diagnoses recorded post-Lyme disease episode only for Lyme disease group

	(I) **Frequency of PTLDS-related diagnoses, Lyme disease group***	(II) **Frequency of PTLDS-related diagnoses, control group†**	(III) χ^2 **test** (of difference in frequencies)	(IV) **Adjusted odds of PTLDS-related diagnoses for Lyme disease group versus control group, (SE) ‡**
**Any PTLDS-related diagnosis**	34.4%	27.6%	p<.001	1.36 (0.01)***
**Any PTLDS-related diagnosis, females**	37.8%	30.1 %	p<.001	1.39 (0.02)***
**Any PTLDS-related diagnosis, males**	30.9%	24.9%	p<.001	1.33 (0.02)***
**Debility and undue fatigue**	13.6%	8.4 %	p<.001	1.69 (0.03)***
**Non-specific signs and symptoms: memory loss, acute/chronic pain**	2.4%	1.2 %	p<.001	1.91 (0.07)***
**Musculoskeletal signs and symptoms**	18.4%	18.9 %	P=.003	0.94 (0.01)***
**Arthropathy**	4.4%	1.8 %	p<.001	2.44 (0.06)***
**Peripheral neuropathy, neuritis**	6.8%	4.6 %	p<.001	1.48 (0.03)***

*** statistically significant at p<.001 level

* Lyme disease sample includes only those persons with a test order and antibiotic treatment, diagnosis and antibiotic treatment, or diagnosis, test order and antibiotic treatment. Lyme disease sample includes only those with 18 consecutive months of enrollment, including 6-month "clean period" of enrollment prior to Lyme disease episode. Lyme disease group PTLDS calculations only include those PTLDS-related diagnoses that were made following a Lyme disease episode, i.e. among the Lyme disease group, PTLDS-related diagnoses made prior to the Lyme disease episode were excluded from the PTLDS analysis.

† Controls were matched to Lyme disease cases on age, sex, region, payer and enrollment year. Control group includes only those with 18 consecutive months of enrollment in a commercial health insurance plan. Control group was restricted to persons with outpatient costs greater than $0.

Lyme disease and control samples are restricted to persons under 65 years of age, with commercial health insurance plans.

‡ Odds are calculated using logistic regression analysis and are adjusted for year, region, age, and sex, and controls for 44 high-cost conditions.
